# Cuticular hydrocarbons for the identification and geographic assignment of empty puparia of forensically important flies

**DOI:** 10.1007/s00414-022-02786-1

**Published:** 2022-02-26

**Authors:** Hannah Moore, Lena Lutz, Victoria Bernhardt, Falko P. Drijfhout, Robert B. Cody, Jens Amendt

**Affiliations:** 1 Defence Academy of the United Kingdom, Cranfield Forensic Institute, Cranfield University, Shrivenham, Wiltshire, SN6 8LA UK; 2 Institute of Legal Medicine, Forensic Biology, University Hospital, Goethe University, Frankfurt am Main, Germany; 3 School of Physical and Geographical Sciences, Keele University, Staffordshire, ST5 5BG UK; 4 JEOL USA, Inc. 11 Dearborn Rd, Peabody, MA 01969 USA

**Keywords:** Cuticular hydrocarbons, GC–MS, PCA, Calliphoridae, Empty puparia, Forensic entomology

## Abstract

Research in social insects has shown that hydrocarbons on their cuticle are species-specific. This has also been proven for Diptera and is a promising tool for identifying important fly taxa in Forensic Entomology. Sometimes the empty puparia, in which the metamorphosis to the adult fly has taken place, can be the most useful entomological evidence at the crime scene. However, so far, they are used with little profit in criminal investigations due to the difficulties of reliably discriminate among different species. We analysed the CHC chemical profiles of empty puparia from seven forensically important blow flies *Calliphora vicina*, *Chrysomya albiceps*, *Lucilia caesar*, *Lucilia sericata*, *Lucilia silvarum*, *Protophormia terraenovae*, *Phormia regina* and the flesh fly *Sarcophaga caerulescens.* The aim was to use their profiles for identification but also investigate geographical differences by comparing profiles of the same species (here: *C. vicina* and *L. sericata*) from different regions. The cuticular hydrocarbons were extracted with hexane and analysed using gas chromatography-mass spectrometry. Our results reveal distinguishing differences within the cuticular hydrocarbon profiles allowing for identification of all analysed species. There were also differences shown in the profiles of *C. vicina* from Germany, Spain, Norway and England, indicating that geographical locations can be determined from this chemical analysis. Differences in *L. sericata*, sampled from England and two locations in Germany, were less pronounced, but there was even some indication that it may be possible to distinguish populations within Germany that are about 70 km apart from one another.

**Supplementary Information:**

The online version contains supplementary material available at 10.1007/s00414-022-02786-1.

## Introduction

Forensic entomology utilises insects that feed on dead tissue and decomposing remains to aid in legal investigations. Possible applications are investigations on mode and circumstances of death, post-mortem modifications of the body or the estimation of the time of death. The latter is performed by analysing the species composition of the necrophagous fauna or by estimating the age of the juvenile insects developing on the dead body. Here, blow flies (Diptera: Calliphoridae) are most important as they often detect and colonise the dead body shortly after death, sometimes only a few hours post mortem. Their age leads to the minimum post mortem interval (PMI_min_), the period between the first insect colonisation and the discovery of the body. Many studies so far have focused on research on fly larvae and pupae and their age determination for the purpose of estimating the PMI_min_ [1–5]. But after 3–4 weeks post mortem, the empty puparia, in which the metamorphosis of the larva via the pupal stage to the adult fly has taken place, are the oldest entomological evidence at the scene and sometimes even the only remnant and evidence of a development that has taken place [6]. Only little research is done on these empty puparia, and currently they are used with little profit in criminal investigations due to the difficulties of reliably discriminate among different species of closely related fly species, or assessing their age as there is no longer any visible change in morphology like the increase in length of the maggots.

However, in the last decade, studies have suggested that invaluable information can be obtained from puparia, and hence new methods for identification and further analyses are being developed [7].

DNA-based techniques show promising results to the field of forensic entomology when it comes to species identification [4, 8–11]. However, while many of these studies focus on juvenile or adult stages of Diptera (and other taxa) or their remains, just a few are dedicated to the identification of empty fly puparia [12]. While this method is promising for fresh material, DNA degradation during the process of ageing can deeply compromise the genetic analysis since the older the fly puparia, the smaller are the amplified DNA fragments [13]. An alternative technique to DNA which has proven its potential of accurately identifying and ageing forensically important species is cuticular hydrocarbon analysis.

Cuticular hydrocarbons (CHC) as a means of species identification has been studied for decades and is used to discriminate between different insect taxa [14, 15]. Their epicuticular wax layer is consisting of hydrocarbons, fatty acids, alcohols, waxes, glycerides, phospholipids and glycolipids. This hydrophobic, flexible layer prevents desiccation as well as penetration of micro-organisms [16]. Hydrocarbons predominate within this layer in many species of insects [13] and are found to be extremely stable [17]. Due to the vast number of different CHC and possible combinations, each species of insect holds its own unique hydrocarbon profile, often referred to as a chemical fingerprint [7, 18, 19].

CHC thus enable the identification of the various developmental stages of insects at the species level, but they can also be used to identify remains and fragments of such stages, like empty puparia, with the main advantage that species identification can not only be established on young, but also on old puparia (due to the stability of hydrocarbons) that have been crushed or deteriorated due to weathering, making the usual morphological characteristics difficult or impossible to visualise under a microscope [7].

The first aim of the present study was to establish the species-specific chemical profiles of the empty puparia from 7 forensically important blow flies and one flesh fly species. The second aim was to then focus on the puparia of two of the blow fly species, *Calliphora vicina* and *Lucilia sericata*, from three different geographical locations to determine whether possible local adaptations impact their chemical profiles and if so, whether this could affect species identification or even, conversely, allow differentiation of local variants.

## Materials and methods

### Insect materials

Empty puparia from 7 forensically important blow flies (*Calliphora vicina*, *Chrysomya albiceps*, *Lucilia caesar*, *Lucilia sericata*, *Lucilia silvarum*, *Phormia regina* and *Protophormia terraenovae*) and one flesh fly (*Sarcophaga caerulescens*) were analysed, thus covering the majority of the first colonisers of the families Calliphoridae and Sarcophagidae found on human cadavers in Europe according to Szpila [20] and Szpila et al. [21]. All species were sampled in Germany, while two blow fly species were additionally collected in England (*C. vicina and L. sericata*) and Norway and Spain (*C. vicina).* For *L. sericata*, different populations within Germany were also analysed (Table 1) from Frankfurt (Germany 1) and Steinau (Germany 2), which are approximately 70 km apart. Empty puparia of all species and populations were obtained by breeding the flies in the laboratory for less than 5 generations. The initial populations or parent generations were established by either baiting the flies in the field or by sampling insect larvae from human bodies during autopsy. Baited or sampled fly larvae were given mixed minced meat (pork and beef) and further reared in the laboratory. Resulting adult flies were held in rearing cages at room temperature (average temperature approximately 20˚C, 79% RH) and a 12:12 L:D cycle. They were provided with water and sugar ad libitum. A piece of fresh pork liver was regularly placed into the cage as a protein source and as oviposítion (or, in the case of the flesh fly *Sarcophaga caerulescens*, as larviposition) medium. Resulting blow fly eggs and flesh fly larvae were transferred separately into an incubator, set between 20 °C ± 1˚C. After 24 h, larvae were transferred from the oviposition medium to mixed minced ad libitum in a plastic cup, which were placed in bigger plastic containers filled with 2 cm of sawdust, serving as the medium for pupariation. After pupariation, every container were checked once per day. After the first fly had hatched, another 3 days were waited, and all empty puparia present up to then were sampled and stored dry at room temperature and 12:12 L:D cycle.

**Table 1:** List of species analysed, sorted according to systematics and origin; 10 specimens per species were analysed, with the exception of *Lucilia sericata* England (*n* = 8) and *Protophormia terraenovae* (*n* = 9)

Species/subpopulation		Country	City/area	Latitude and longitude
**Sarcophagidae**				
**1**	*Sarcophaga caerulescens* Zetterstedt, 1838	Germany	Hammelburg	50°05′N, 9°86′E
**Calliphoridae**				
**Calliphorinae**				
**2**	*Calliphora vicina* Robineau-Desvoidy, 1830	England	Keele University	53°013′N, 2° 17′W
**3**	*Calliphora vicina*	Germany	Frankfurt/Main	50°11′N, 8°66′E
**4**	*Calliphora vicina*	Norway	Ballstad	68°05′N, 13°33′E
**5**	*Calliphora vicina*	Spain	Villaviciosa de Odón	40°21′N, 3°54′W
**Luciliinae**				
**6**	*Lucilia caesar* (Linnaeus, 1758)	Germany	Hannover	52°39′N, 9.69′E
**7**	*Lucilia sericata* (Meigen, 1826)	England	Keele University	53°01′N, 2° 17′W
**8**	*Lucilia sericata*	Germany 1	Frankfurt/Main	50°11′N, 8°66′E
**9**	*Lucilia sericata*	Germany 2	Steinau	50°31′N, 9°46′E
**10**	*Lucilia silvarum* (Meigen, 1826)	Germany	Hammelburg	50°05′N, 9°86′E
**Chrysomyiinae**				
**11**	*Chrysomya albiceps* (Wiedemann, 1819)	Germany	Frankfurt/Main	50°11′N, 8°66′E
**12**	*Phormia regina* (Meigen, 1826)	Germany	Frankfurt/Main	50°11′N, 8°66′E
**13**	*Protophormia terraenovae (*Robineau-Desvoidy, 1830)	Germany	Frankfurt/Main	50°11′N, 8°66′E

### Sample preparation

For each sample (*n* = 10), two puparia were used. They were placed into a 2 mL GC vial and submerged with hexane (350 μL) for 10 to 15 min. The hexane extract was collected in a clean 2-mL vial and then left to evaporate until the extract could be transferred to a 300 μL flat bottomed insert and left to dry down completely. All samples were stored dry in the refrigerator at 4 ˚C until they were required for analysis. The dried extract was then reconstituted in 30 μL of hexane before GC–MS analysis which was carried out using the autosampler.

### Chemical analysis of extracts

Chemical analysis of all extracts was carried out on an Agilent Technologies 6890 N Network GC with a split/splitless injector at 250 °C, a Restek Rxi-1MS capillary column (30 m × 0.25 mm ID, 0.25 μm film thickness) and coupled to an Agilent 5973 Network Mass Selective Detector. The GC was coupled to a computer and data processed with Agilent Chemstation software. Elution was carried out with helium at 1 mL/min. The oven temperature was programmed to be held at 50 °C for 2 min and then ramped to 200 °C at 25 °C/min, then from 200 to 260 °C at 3 °C/min and finally from 260 to 320 °C at 20 °C/min where it was held for 2 min. The mass spectrometer was operated in Electron Ionisation mode at 70 eV, scanning from 40 to 500 amu at 1.5 scans s^−1^. Hydrocarbons were identified using a library search (NIST08), the diagnostic fragmented ions and the Kovats indices. Individual chromatograms were exported to text files as peak lists containing retention times and peak areas. The identified hydrocarbons were manually aligned based on their retention times and mass spectra.

### Statistical analysis

Chemometric analysis was carried out with Mass Mountaineer software as described in a previous publication [22]. For analysis, the largest peak area in each sample was assigned as 100%, and individual peak areas were normalised to the sum of all peak areas for the selected compounds in each sample. Fifty-three statistically significant compounds were selected by calculating analysis of variance (ANOVA) for each compound between the two classes that showed the greatest difference in means. Peaks with a *p* value greater than 0.05 were omitted from the statistical analysis (Table 2).

**Table 2:** List of the compounds extracted from empty puparia of seven species with the total percentage of each compound present, ± the percentage standard deviation for each species

			*S. caerulescens*	*C. vicina*	*C. vicina*	*C. vicina*	*C. vicina*	*L. caesar*	*L. sericata*	*L. sericata*	*L. sericata*	*L. silvarum*	*Ch. albiceps*	*P. regina*	*P. terraenovae*
			Germany	England	Germany	Norway	Spain	Germany	England	Germany 1	Germany 2	Germany	Germany	Germany	Germany
			*n* = *10*	*n* = *10*	*n* = *10*	*n* = *10*	*n* = *10*	*n* = *10*	*n* = *8*	*n* = *10*	*n* = *10*	*n* = *10*	*n* = *10*	*n* = *10*	*n* = *9*
Pk no	Ret Time	Pk ID	%	%	%	%	%	%	%	%	%	%	%	%	%
1	12.280	C18	ND	ND	ND	ND	ND	ND	ND	ND	ND	ND	ND	1.83 ± 3.51	ND
2	14.471	C21	ND	ND	ND	ND	ND	ND	ND	ND	ND	ND	ND	ND	0.46 ± 0.59
3	17.302	2-Methyl C22	ND	ND	ND	ND	0.36 ± 0.60	ND	ND	ND	ND	ND	ND	ND	ND
4	17.991	C23	ND	ND	0.95 ± 1.19	0.50 ± 0.46	1.24 ± 1.95	ND	ND	ND	ND	ND	ND	1.53 ± 1.02	6.13 ± 6.12
5	18.734	9-Methyl C23	ND	ND	ND	ND	0.27 ± 0.46	ND	ND	ND	ND	ND	ND	ND	ND
6	19.427	3-Methyl C23	ND	ND	ND	ND	0.20 ± 0.32	ND	ND	ND	ND	ND	ND	ND	ND
7	19.772	C24	ND	0.76 ± 0.64	ND	ND	ND	ND	ND	ND	ND	ND	ND	ND	ND
8	21.340	2-Methyl C24	ND	ND	ND	ND	2.69 ± 5.12	ND	ND	ND	ND	ND	ND	ND	ND
9**	21.487	C25:1	ND	ND	ND	ND	5.70 ± 10.11	ND	ND	ND	ND	ND	ND	ND	ND
10	22.129	C25	ND	4.09 ± 4.80	11.93 ± 11.02	6.16 ± 6.10	6.91 ± 8.20	2.97 ± 3.15	3.88 ± 5.08	2.45 ± 3.01	2.08 ± 2.55	0.90 ± 0.96	3.40 ± 3.13	4.19 ± 3.36	11.10 ± 11.27
11	22.829	9 + 11 + 13-Methyl C25	ND	0.95 ± 1.19	ND	ND	2.69 ± 4.67	ND	ND	ND	ND	ND	ND	ND	ND
12	22.958	7-Methyl C25	ND	ND	ND	ND	0.24 ± 0.30	ND	ND	ND	ND	ND	ND	ND	ND
13	23.139	5-Methyl C25	ND	ND	ND	ND	0.25 ± 0.30	ND	ND	ND	ND	ND	ND	ND	ND
14	23.635	3-Methyl C25	ND	ND	4.11 ± 4.37	0.54 ± 0.79	0.70 ± 0.76	ND	ND	ND	ND	ND	ND	ND	0.65 ± 0.58
15	24.193	C26	ND	2.72 ± 2.42	2.14 ± 2.02	1.84 ± 1.86	1.19 ± 0.68	1.64 ± 1.55	1.41 ± 1.82	1.44 ± 1.66	1.20 ± 1.47	1.17 ± 0.98	ND	1.41 ± 1.18	1.88 ± 1.99
16	24.364	x,7-DiMethyl C25	ND	ND	0.86 ± 0.83	ND	ND	ND	ND	ND	ND	ND	ND	ND	ND
17	24.909	12,14,16-Methyl C26	ND	ND	ND	ND	0.46 ± 0.66	ND	ND	ND	ND	ND	ND	ND	ND
18	25.536	2-Methyl C26	ND	0.84 ± 0.73	1.21 ± 1.41	0.76 ± 1.05	ND	ND	ND	ND	ND	0.55 ± 0.83	ND	ND	ND
19**	25.755	C27:1	ND	ND	ND	ND	9.11 ± 17.91	ND	ND	ND	ND	ND	ND	ND	ND
20**	25.944	C27:1	ND	ND	ND	ND	0.75 ± 0.92	ND	ND	ND	ND	ND	ND	ND	ND
21**	26.037	C27:1	ND	ND	ND	ND	1.49 ± 2.67	ND	ND	ND	ND	ND	ND	ND	ND
22	26.512	C27	23.31 ± 20.59	40.53 ± 34.56	36.14 ± 30.87	44.89 ± 38.70	26.87 ± 14.25	47.94 ± 49.83	34.05 ± 39.39	43.81 ± 47.94	40.25 ± 44.01	42.00 ± 32.26	5.07 ± 4.74	32.83 ± 26.77	43.73 ± 43.17
23	27.077	9 + 11 + 13-Methyl C27	4.29 ± 5.74	3.58 ± 5.44	5.90 ± 6.17	3.36 ± 5.49	3.69 ± 3.24	0.77 ± 0.66	ND	ND	0.71 ± 0.91	2.14 ± 2.54	ND	3.88 ± 3.73	0.98 ± 1.32
24***	26.799	9-Methyl C27	ND	0.81 ± 0.79	ND	ND	ND	ND	ND	ND	ND	ND	ND	ND	ND
25	27.251	7-Methyl C27	2.39 ± 2.50	1.35 ± 1.58	2.07 ± 2.18	0.99 ± 1.65	0.84 ± 0.67	ND	ND	ND	ND	0.68 ± 0.67	ND	1.20 ± 1.09	ND
26***	27.363	11,15-DiMethyl C27	ND	1.07 ± 1.27	ND	ND	ND	ND	ND	ND	ND	ND	ND	ND	ND
27	27.443	5-Methyl C27	1.19 ± 1.14	0.99 ± 1.10	1.69 ± 1.92	0.95 ± 1.52	0.78 ± 0.63	ND	ND	ND	ND	ND	ND	ND	ND
28	27.684	9,13-DiMethyl C27	0.93 ± 0.82	ND	ND	ND	ND	ND	ND	ND	ND	ND	ND	2.14 ± 2.22	ND
29	28.002	3-Methyl C27	10.19 ± 11.08	7.71 ± 7.02	10.47 ± 11.01	7.13 ± 9.06	4.97 ± 3.52	5.64 ± 5.86	4.26 ± 5.49	3.54 ± 3.76	4.42 ± 5.06	4.53 ± 5.24	3.34 ± 3.19	8.56 ± 7.23	2.91 ± 3.92
30	28.111	5,x-DiMethyl C27	ND	1.19 ± 1.58	1.28 ± 1.47	1.13 ± 1.66	0.77 ± 0.60	ND	ND	ND	ND	ND	ND	ND	ND
31***	28.324	TriMethyl C27	ND	1.31 ± 1.43	ND	ND	ND	ND	ND	ND	ND	ND	ND	ND	ND
32	28.437	C28	2.12 ± 2.12	2.32 ± 2.66	1.08 ± 1.17	3.24 ± 3.51	2.63 ± 1.59	4.07 ± 4.31	2.95 ± 2.70	3.52 ± 3.90	2.66 ± 2.55	2.92 ± 2.88	1.18 ± 0.76	2.64 ± 2.55	2.23 ± 2.45
33	28.577	x,7-DiMethyl C27	1.22 ± 1.20	ND	1.87 ± 1.91	1.65 ± 2.24	1.09 ± 0.85	ND	ND	ND	ND	1.18 ± 2.08	ND	ND	ND
34	28.995	12,14,16-Methyl C28	ND	0.97 ± 1.32	0.74 ± 1.09	0.80 ± 1.26	0.74 ± 0.69	ND	ND	ND	ND	ND	ND	1.20 ± 1.24	ND
35***	28.782	8-Methyl C28	ND	1.04 ± 1.07	ND	ND	ND	ND	ND	ND	ND	ND	ND	ND	ND
36**	29.271	C29:2	ND	ND	ND	ND	1.13 ± 1.88	ND	ND	ND	ND	ND	ND	ND	ND
37	29.321	2-Methyl C28	ND	2.74 ± 2.53	2.68 ± 3.36	1.77 ± 2.50	2.06 ± 1.81	3.91 ± 4.23	1.62 ± 3.00	1.94 ± 1.94	2.03 ± 1.58	3.19 ± 3.17	9.48 ± 9.54	2.83 ± 2.98	ND
38***	29.500	C29:1	ND	ND	ND	ND	1.92 ± 2.62	ND	0.90 ± 2.14	ND	ND	ND	ND	ND	ND
39	29.458	6,x-DiMethyl C28 (x = 10,12,14)	ND	1.83 ± 2.46	1.02 ± 1.45	1.14 ± 1.80	ND	ND	ND	ND	ND	ND	ND	ND	ND
40	29.670	x,14-DiMethyl C28	ND	ND	1.01 ± 1.48	ND	ND	ND	ND	ND	1.06 ± 1.00	3.69 ± 4.77	ND	ND	ND
41	29.744	C29	30.47 ± 24.32	10.81 ± 11.60	5.27 ± 5.81	15.70 ± 10.85	10.93 ± 5.51	20.99 ± 19.03	31.99 ± 18.69	32.64 ± 26.71	29.72 ± 21.54	18.09 ± 19.99	12.70 ± 10.60	12.17 ± 14.01	19.16 ± 18.10
42	29.900	4,8,12-TriMethyl C28	ND	1.22 ± 1.35	0.66 ± 0.80	ND	0.51 ± 0.46	ND	ND	ND	ND	ND	ND	ND	ND
43	30.036	9 + 11-Methyl C29	7.89 ± 10.59	5.08 ± 5.52	3.28 ± 4.28	3.17 ± 4.56	2.85 ± 2.36	5.37 ± 4.48	3.23 ± 4.68	4.01 ± 2.24	5.77 ± 7.38	8.02 ± 9.28	2.44 ± 2.13	4.64 ± 6.16	2.15 ± 1.76
44***	30.060	9-Methyl C29	ND	ND	ND	ND	ND	ND	1.12 ± 1.53	1.12 ± 0.74	1.69 ± 1.76	2.47 ± 3.11	ND	2.92 ± 3.54	ND
45	30.119	7-Methyl C29	2.25 ± 2.82	1.10 ± 1.19	0.90 ± 0.94	1.04 ± 1.05	0.85 ± 0.61	1.36 ± 1.04	0.71 ± 0.97	1.00 ± 0.88	1.51 ± 1.59	2.16 ± 1.74	0.99 ± 0.50	2.71 ± 3.39	1.43 ± 1.13
46	30.235	5-Methyl C29	2.01 ± 2.52	0.57 ± 0.54	0.84 ± 1.12	0.97 ± 1.05	0.83 ± 0.54	0.97 ± 1.01	ND	ND	ND	ND	1.05 ± 0.50	1.57 ± 2.00	0.88 ± 0.90
47***	30.146	11 + 15-DiMethyl C29	ND	0.47 ± 0.49	ND	ND	ND	ND	ND	ND	ND	ND	ND	ND	ND
48	30.326	9,13 + 9,17-DiMethyl C29	ND	0.70 ± 0.83	0.92 ± 1.04	ND	ND	0.73 ± 0.78	0.63 ± 0.71	0.78 ± 0.55	1.23 ± 1.58	1.60 ± 1.88	ND	3.73 ± 4.68	ND
49	30.395	3-Methyl C29	5.02 ± 6.32	1.36 ± 1.37	0.96 ± 1.08	1.42 ± 1.60	1.11 ± 0.87	1.36 ± 1.50	0.83 ± 0.96	0.75 ± 0.91	1.11 ± 1.57	1.06 ± 1.01	3.87 ± 2.59	5.43 ± 6.10	2.83 ± 2.94
50	30.446	5,17-DiMethyl C29	1.93 ± 2.20	ND	ND	ND	ND	ND	ND	ND	ND	ND	ND	ND	ND
51	30.596	C30	0.79 ± 0.96	0.38 ± 0.34	ND	0.22 ± 0.27	ND	ND	1.26 ± 0.95	0.77 ± 0.68	0.61 ± 0.88	ND	0.58 ± 0.55	ND	0.39 ± 0.58
52	30.895	C28:Ald	ND	ND	ND	0.29 ± 0.30	0.23 ± 0.19	ND	ND	ND	ND	0.79 ± 1.96	1.57 ± 1.92	ND	ND
53	31.031	2-Methyl C30	ND	ND	ND	ND	0.52 ± 0.83	0.63 ± 0.80	ND	0.80 ± 1.15	0.76 ± 0.87	0.74 ± 0.89	10.34 ± 13.00	0.63 ± 0.63	ND
54	31.146	C31:1	ND	0.42 ± 0.65	ND	ND	ND	ND	ND	ND	ND	ND	2.22 ± 2.58	ND	ND
55	31.225	x,14-DiMethyl C30	ND	ND	ND	ND	ND	0.83 ± 1.04	ND	0.96 ± 3.40	0.51 ± 0.51	ND	3.62 ± 4.00	ND	ND
56	31.280	C31	2.86 ± 3.08	0.73 ± 0.99	ND	0.32 ± 0.66	0.24 ± 0.38	ND	8.32 ± 8.37	ND	2.69 ± 3.20	1.64 ± 3.33	2.49 ± 2.14	ND	0.88 ± 1.32
57	31.485	11,13-Methyl C31	1.16 ± 1.99	ND	ND	ND	0.18 ± 0.26	0.81 ± 0.75	0.95 ± 1.57	0.48 ± 0.54	ND	0.48 ± 0.43	14.97 ± 13.38	1.96 ± 2.60	1.58 ± 1.34
58	31.866	C32	ND	0.26 ± 0.36	ND	ND	ND	ND	1.00 ± 0.86	ND	ND	ND	ND	ND	ND
59	32.574	C33	ND	0.11 ± 0.19	ND	ND	ND	ND	0.88 ± 1.08	ND	ND	ND	ND	ND	ND
60***	32.457	C33:1	ND	ND	ND	ND	ND	ND	ND	ND	ND	ND	17.10 ± 21.42	ND	ND
61	32.900	11,13,15,17-Methyl C33	ND	ND	ND	ND	ND	ND	ND	ND	ND	ND	3.58 ± 3.32	ND	0.63 ± 0.55

*Tentative identification based on Kovats Index values and match with NIST08 Library database.

**Double bond position not assigned.

***Not selected for statistical analysis.

*ND* not detected.

## Results

### CHC profiles

The empty puparia of the seven blow flies and one flesh fly species yielded chemical profiles of 61 peaks with percentage areas exceeding 0.5% of the total. The chemical profiles consisted of *n*-alkanes (21%), alkenes (13%), methyl branched hydrocarbons (64%) and unknowns (1%) with the chain length ranging from C18:H to C33:H (Table 2). For this study, the double bond positions were not determined for the alkenes and alkadienes. In general, the odd numbered *n*-alkanes yielded significantly larger peak areas, with heptacosane (C27:H, peak 22) dominating the profiles in most species, followed by nonacosane (C29:H, peak 41). The most dominant methyl branched hydrocarbon was 3-methylheptacosane.

*Calliphora vicina* from Spain had the largest number of alkenes within its profile (10%). A number of these alkenes were observed in the Spanish specimens only (i.e. not observed in *C. vicina* from Norway, Germany or England). Moreover, *C. vicina* from Spain revealed several other geographically specific compounds, such as peaks 3, 5, 6, 8, 9, 12, 13, 17, 19, 20 and 21 (Table 1).

*Calliphora vicina* from England revealed two geographically specific compounds which were tetracosane (24:H, peak 7) and 11 + 15-dimethyl nonacosane (peak 47). The profile of *C. vicina* Germany was the only one to contain x,7-dimethyl pentacosane (peak 16). Distinctions between *C. vicina* by geographical origin can be seen in the principal component analysis plot shown in Figure S2.

*Phormia regina* had a species-specific compound which was octadecane (C18:H, peak 1)) and *P. terraenovae* has a compound unique to its chemical profile of heneicosane C21:H (peak 2). Tritriacontene (C33:1) was only observed in *Ch. albiceps* and the two species from England (*C. vicina* and *L. sericata*) both shared two compounds in common, dotriacontane (C32:H, peak 58)) and tritriacontane (C33:H, peak 59), implying that they were geographically specific but not species specific.

In general, the three geographical sets of *L. sericata* (England, Germany 1, Germany 2) were quite similar, sharing a lot of compounds within their chemical profiles. However, noticeable differences were detected. Germany 2 was the only one of the three geographical locations to yield 9 + 11 + 13-Methyl C27 (peak 23). *L. sericata* from England was the only geographical region of the three to detect an alkene within its profile (C29:1, Peak 38), while peak 55 (x, 14-DiMethyl C30 was detectable in both Germany 1 and 2 and not in the England samples, implying that they were geographically specific but not species specific. The higher chain length n-alkanes (C31, C32 and C33) were all detectable in *L. sericata* England; however, of the three alkanes, only C31 was detectable in Germany 2, and none were detectable in Germany 1, making C32 and C33 geographically specific. Distinctions between *L. sericata* by geographical origin can be seen in the principal component analysis plot shown in Figure S3.

### Chemical identification

**Fig. 1:**
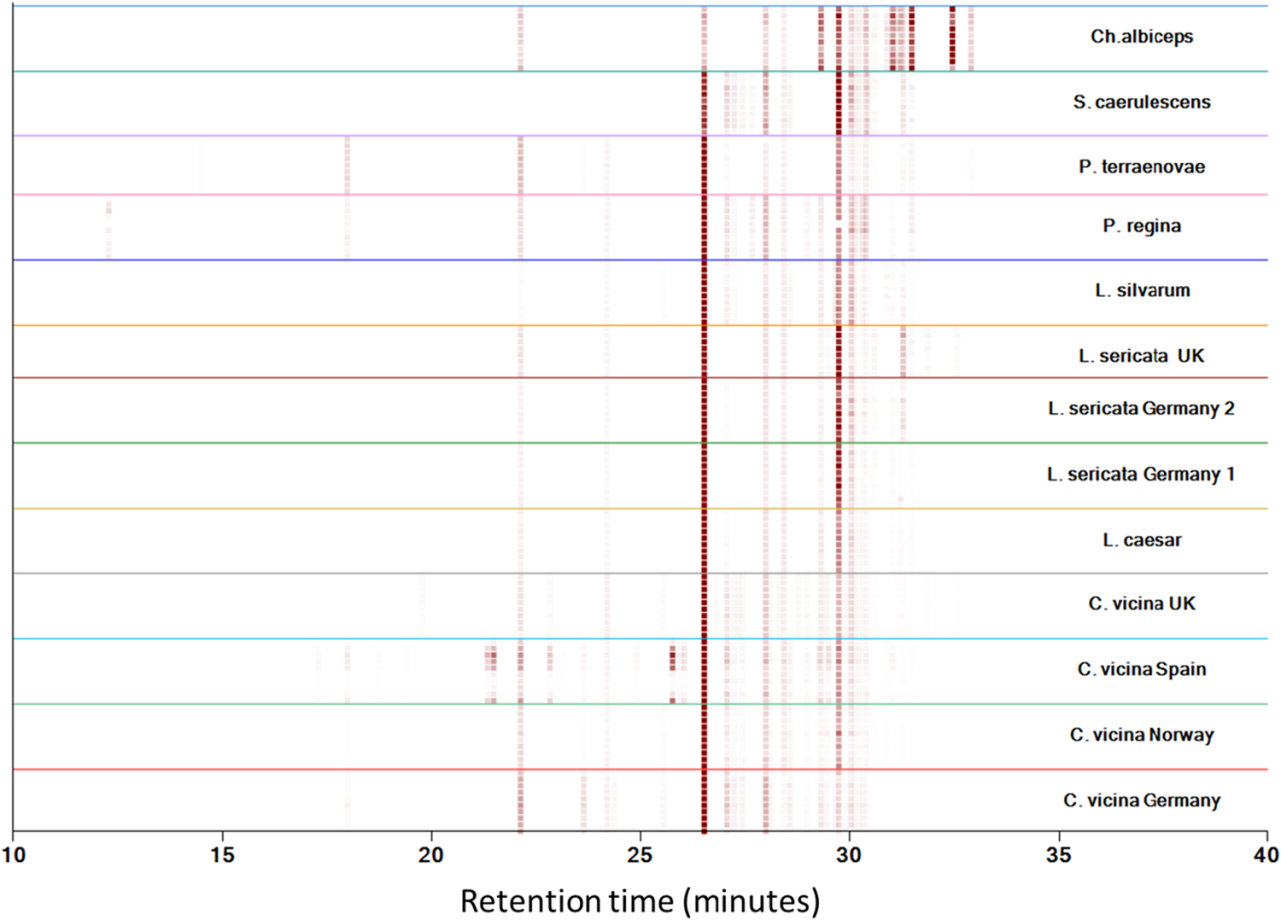
Heat map of all 61 compounds from the 8 species (thirteen data sets), showing species-dependent and geographical-dependent differences in the chromatograms. The *x*-axis represents the retention time, and the chromatographs are grouped along the *y*-axis by species

All chromatograms are displayed as a heat map in Fig. 1. The heat map is a visual aid, enabling multiple chromatographs to be efficiently stacked and grouped by species and geographic origin for comparison in a small vertical space, in which darker spots represent larger peak areas. For example, the most abundant compound, with a retention time of around 26.5 min on the heat map, is C27 (Table 2, peak number 22). The pattern valid for the corresponding species or its geographical origin is located under the respective coloured line with results from up to 10 individual replicate samples. The compounds used for classifying are presented in Table 2.

**Fig. 2:**
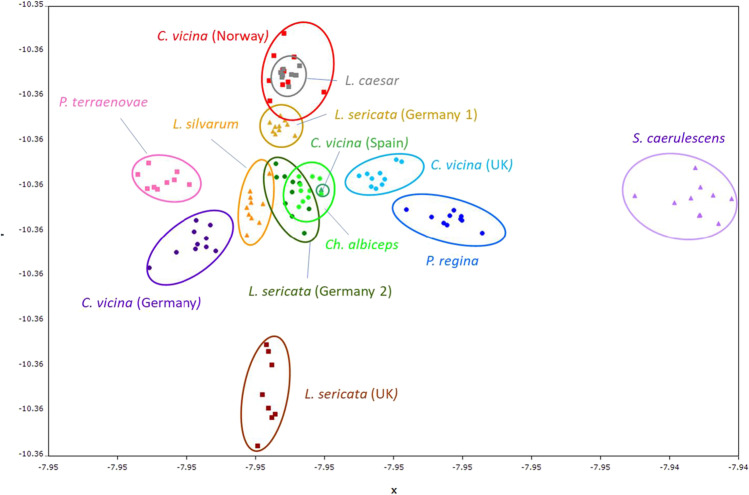
Linear discriminant analysis (LDA) showing clearer visual separation between classes

As an unsupervised method, the principal component analysis (PCA) was carried out to determine whether there are sufficient chemical differences between classes to justify further analysis. PCA calculated using the correlation matrix (Figure S4) shows clustering for members of each class, with each class assigned a different colour. However, the separation between class members in this figure is difficult to clearly visualise. The supervised learning method linear discriminant analysis (LDA) shows a visually clearer separation between classes (Fig. 2).

Although LDA already showed visual separation between classes, support vector machine (SVM) classification was chosen as the most efficient classifier. SVM is a supervised learning method that does not produce a graphical display, but which is a highly effective classifier. Leave-one-out cross validation (LOOCV) with SVM gave 100% classification accuracy. Additional validation was carried out by omitting 30 percent of the samples from the training set to be treated as “unknowns.” SVM classification correctly identified the genus, species and geographic origin of 100% of the “unknowns” (Table S1).

## Discussion

Chemotaxonomy within the field of entomology has been around for many years, and it is widely accepted that CHC analysis provides an excellent means of species identification across a broad range of insect orders like Hymenoptera [15, 23–32], Isoptera [14, 33], Blattodea [31, 34, 35] and Coleoptera [28–30]. CHC analysis has also been used for Diptera [7, 22, 31, 36–39], providing a complementary technique when the taxonomical identification is ambiguous or even not feasible, which could be due to the damaged physical condition or DNA degradation, or quite simply because the morphology between particular species is too similar to identify them [22, 40].

Necrophagous flies are the most important indicators in forensic entomology as they provide a wealth of information within an investigation, from evidence of neglect of living persons or persons who have died because of it, over toxicological histories of deceased persons to the determination of a PMI_min_ [41]. Moreover, possible geographical variability of single species could provide information whether or not the victim had been relocated from the site at which death occurred [42].

A number of papers have begun to explore the potential of using CHC for species identification or population assignment and ageing various life stages of forensically important Calliphoridae [22, 36, 43–47].

Byrne et al. [48] studied the chemical changes between different geographical populations of the black blow fly, *Phormia regina*. Populations from three locations were examined and using GC–MS analysis to analyse and identify the CHCs; they were able to successfully distinguish between the different locations. Brown et al. [49] examined the CHC compositions of male and female *Chrysomya bezziana* from 15 different locations covering Africa, the Middle East, India, Southeast Asia and Papua New Guinea. Due to the fact this species is known to be a parasite of warm-blooded animals, tracking their geographical location to determine the origin of flies is very important. Their results showed qualitative similarities but quantitative differences, allowing for the differentiation between the geographical locations. Ye et al. [7] examined the chemical composition from six necrophagous flies to determine their taxonomic differentiation. They were able to chemically distinguish all species under controlled laboratory conditions. Moore et al. [22] were the first to carry out an extensive study on the identification of 11 species of Sarcophagidae (males and females) from dry pinned museum samples. This family of Diptera can be notoriously challenging to taxonomically identify, and the results presented in this paper are especially relevant for the flesh fly females, which are known to be more difficult to identify than males using morphological criteria.

Just a few studies are addressing empty puparia for identification so far and looked also at the difference of geographical location and how the local climate or habitat might alter the chemical profiles of the necrophagous flies and/or their puparia.

Braga et al. [38] successfully examined the cuticular hydrocarbon profiles of four species of Sarcophagidae of forensic importance in South America—*Peckia chrysostoma* (Wiedemann), *P. intermutans* (Walker), *P. lambens* (Wiedemann) *and Sarcophaga ruficornis* (Fabricius)—using empty puparia. The specimens were reared in the laboratory in a controlled environment and analysed by using GC–MS and. By applying Bray–Curtis distances to the data sets, Braga et al. could successfully discriminate between all four species. Musah et al. [50] examined species classification from chemical fingerprint signatures using direct analysis in real time (DART) mass spectrometry. This method was applied to a variety of species which included endangered woods, biodiesel feedstocks, psychoactive plant products and *Eucalyptus*. It was also successfully applied to empty puparia of *Chrysomya rufifacies*, *Lucilia sericata*, *L. cuprina* and *Cochliomyia macellaria* allowing for these species to be chemically distinguishable from their CHC profiles.

As with adult and immature stages, morphology and DNA are options for identifying puparia of forensically important Diptera. But due to the facts that in puparia the number of helpful diagnostic features at species level is significantly lower than in adults, that they are more difficult to recognise than in larvae due to their dark colouration and that, depending on the crime scene and time of storage, they are often covered with dust and dirt, which obscure the diagnostic features, a correct identification of the specimens is difficult or even impossible and requires sufficient experience [51]. DNA might be a useful alternative, since genotyping can be quick and simple compared to morphological analysis of specimens and the time-consuming rearing procedure to obtain adult specimens for identification. The costs of DNA analysis for species identification are negligible in a forensic laboratory, as are possible time aspects. But Mazzanti et al. [52] highlighted some potential pitfalls in DNA based puparia identification like DNA degradation, unsuccessful amplification and contamination. DNA is hard to get from such specimens due to its small amount and the many disturbing chemical components in the puparia. In fact, serious publications on this topic hardly exist. However, recently Pradelli et al. [51] successfully extracted and identified DNA of the blow fly *L. sericata* from dirty puparia cleaned by different chemical methods. But such results need not necessarily be the rule due to the low amount of tissue in a single puparium suitable to extract nucleic acids, and it is therefore important to use complementary and supportive methods.

We showed in the present study that cuticular hydrocarbon analysis is such a method, which can also provide further information. For future studies, it is important to include more taxa (e.g. the important family Muscidae [53]) and to better map intraspecific variability and understand its causes. This would not only validate a basic framework of important CHCs, but perhaps even establish these chemical elements as markers for e.g. stress during the larval growth and metamorphosis. As CHCs are an important communication tool, their presence and amount could indicate e.g. an interaction with competitive species on the diet, and a varying composition and concentration could be an indication of drought stress during the pupal phase. Before reaching this point, however, further studies are necessary to determine the function of individual, potential marker CHCs.

## Supplementary Information

Below is the link to the electronic supplementary material.

Supplementary file1 (DOCX 1077 KB)
