# Supplementary material for: Cuticular hydrocarbons for the identification and geographic assignment of empty puparia of forensically important flies
Source: Int J Legal Med. 2022 Feb 26;136(6):1791–800. doi: 10.1007/s00414-022-02786-1 (PMC9576650; doi:10.1007/s00414-022-02786-1)
Supplement: Supplementary file 1 — Supplementary file1 (DOCX 1077 KB) [file 414_2022_2786_MOESM1_ESM.docx]

**Supplementary Data**

Cuticular hydrocarbons for the identification and geographic assignment of empty puparial cases of forensically important flies

Hannah Moore^a*^, Lena Lutz^b^, Vicky Bernhardt^b^, Falko P. Drijfhout^c^, Robert B. Cody^d^, Jens Amendt^b^

*^a^Cranfield Forensic Institute, Cranfield University, Defence Academy of the United Kingdom, Shrivenham, Wiltshire, SN6 8LA, UK*

*^b^* *Institute of Legal Medicine, Forensic Biology, University Hospital, Goethe University Frankfurt am Main*

*^c^School of Physical and Geographical Sciences, Keele University, Staffordshire, ST5 5BG, UK*

*^d^JEOL USA, Inc. 11 Dearborn Rd., Peabody MA 01969 USA*

**corresponding author:* [*h.e.moore@cranfield.ac.uk*](mailto:h.e.moore@cranfield.ac.uk)

5.00

10.00

15.00

20.00

25.00

30.00

0

5000000

1e+07

1.5e+07

2e+07

2.5e+07

3e+07

3.5e+07

4e+07

4.5e+07

5e+07

5.5e+07

6e+07

6.5e+07

7e+07

7.5e+07

8e+07

8.5e+07

9e+07

9.5e+07

Time-->

Abundance

*Ch. albiceps*

*L. caesar*

*P. regina*

*S. caerulescens*

*L. sericata* Germany I

*L. sericata Germany II*

*L. silvarum*

*P. terraenovae*

C. vicina Germany

*C. vicina* Norway

*C. vicina* Spain

Figure S1: GC chromatogram overlay of the chemical profiles of all species and geographical origin.

Table S1: Compounds used for the principal component analysis (PCA), leave-one-out cross validation (LOOCV) and support vector machine (SVM) classification.

| **Retention Time** | **Compound** |
| --- | --- |
| 12.28 | C18; |
| 14.471 | C21; |
| 17.302 | 2MeC22; |
| 17.991 | C23; |
| 18.734 | 9MeC23; |
| 19.427 | 3MeC23; |
| 19.772 | C24; |
| 21.34 | 2MeC24; |
| 21.487 | C25Alkene; |
| 22.129 | C25; |
| 22.829 | 9+11+13MeC25; |
| 22.958 | 7MeC25; |
| 23.139 | 5MeC25; |
| 23.635 | 3MeC25; |
| 24.193 | C26; |
| 24.364 | X,7diMeC25; |
| 24.909 | 12,14,16MeC26; |
| 25.536 | 2MeC26; |
| 25.755 | C27 Alkene; |
| 25.944 | C27 Alkene; |
| 26.037 | C27 Alkene; |
| 26.512 | C27; |
| 27.077 | 9+11+13MeC27; |
| 27.251 | 7MeC27; |
| 27.443 | 5MeC27; |
| 27.684 | 9,13diMeC27; |
| 28.002 | 3MeC27; |
| 28.111 | 5,XdiMeC27; |
| 28.437 | C28; |
| 28.577 | X,7diMeC27; |
| 28.995 | 12,14,16MeC28; |
| 29.271 | AlkadieneC29; |
| 29.321 | 2MeC28; |
| 29.5 | C29 Alkene; 6,XdiMeC28 (X = 10,12,14); |
| 29.67 | X,14diMeC28; |
| 29.744 | C29; |
| 29.9 | 4,8,12triMeC28; |
| 30.036 | 9+11MeC29; 9MeC29; |
| 30.119 | 7MeC29; 11+15-diMethylnonacosane; |
| 30.235 | 5MeC29; |
| 30.326 | 9,13+9,17diMeC29; |
| 30.395 | 3MeC29; |
| 30.446 | 5,17diMeC29; |
| 30.596 | C30; |
| 30.895 | C28:Ald; |
| 31.031 | 2MeC30; |
| 31.146 | C31Alkene?; |
| 31.225 | X,14diMeC30; |
| 31.28 | C31; |
| 31.485 | 11,13MeC31; |
| 31.866 | Doctriacontane; |
| 32.574 | Tritriacontane; |
| 32.9 | 11,13,15,17MeC33; |


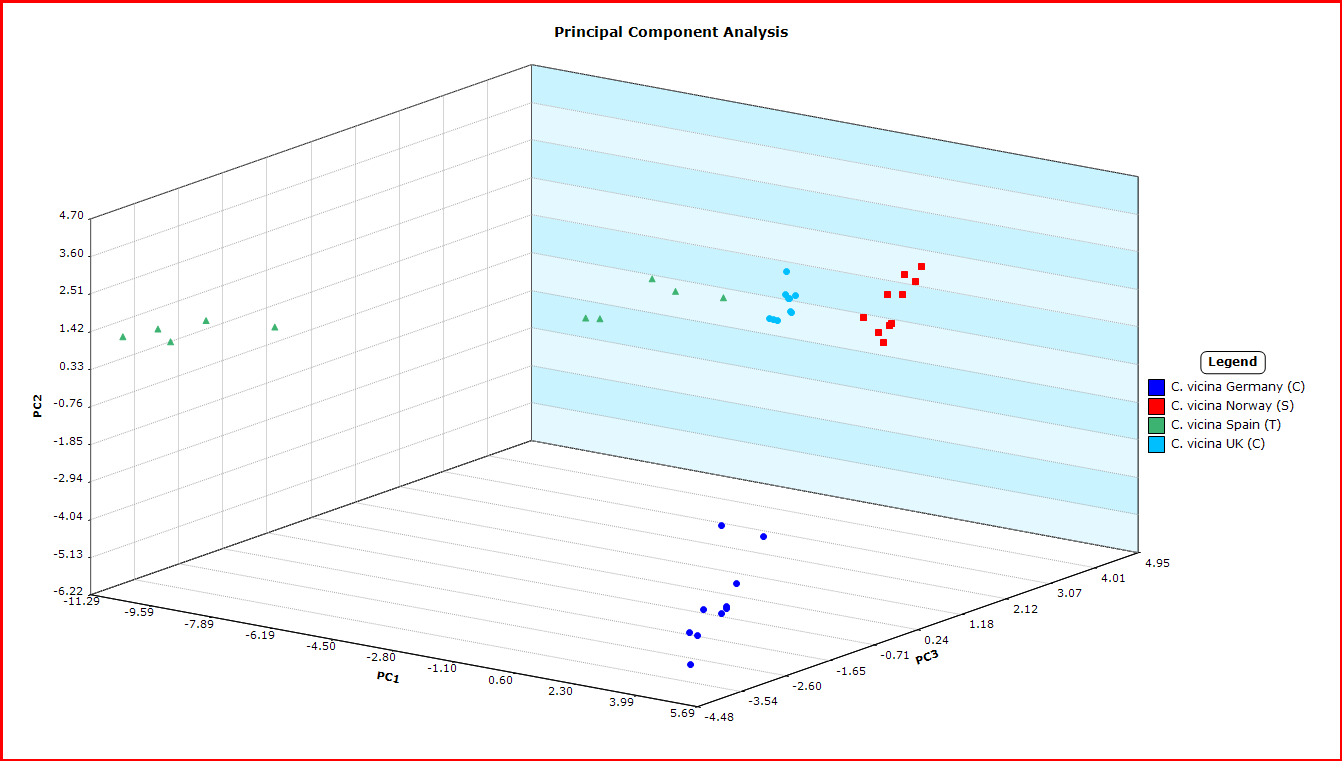


Figure S2: Principal component analysis for from *C. vicina* by geographical origin. The compounds used for PCA are given in Table 1. The first three principal components cover 40.2%, 22.8% and 18.5% of the variance respectively for a total of 81.5% variance. Factor loadings for the first two principal components are shown in the biplot (Figure S3).

Figure S3. Biplot showing PCA factor loadings for *C. vicina* by geographic origin.


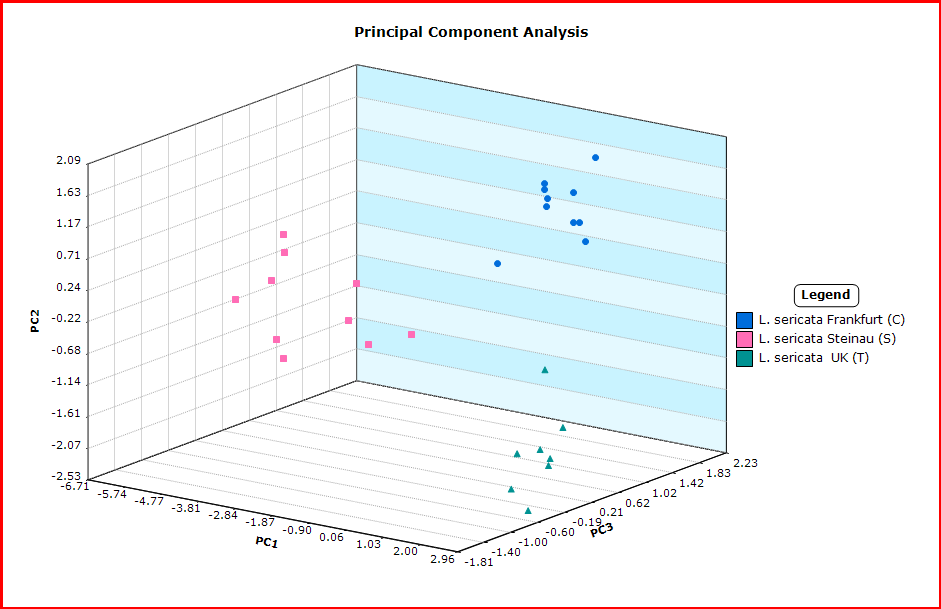


Figure S4: Principal component analysis for from *L. sericata* by geographical origin. The compounds used for PCA are given in Table 1. The first three principal components cover 59.2%, 19.8% and 11.0% of the variance respectively for a total of 90.0% variance. Factor loadings for the first two principal components are shown in the biplot (Figure S5)..

Figure S5. Biplot showing PCA factor loadings for *L. sericata* by geographic origin.

*
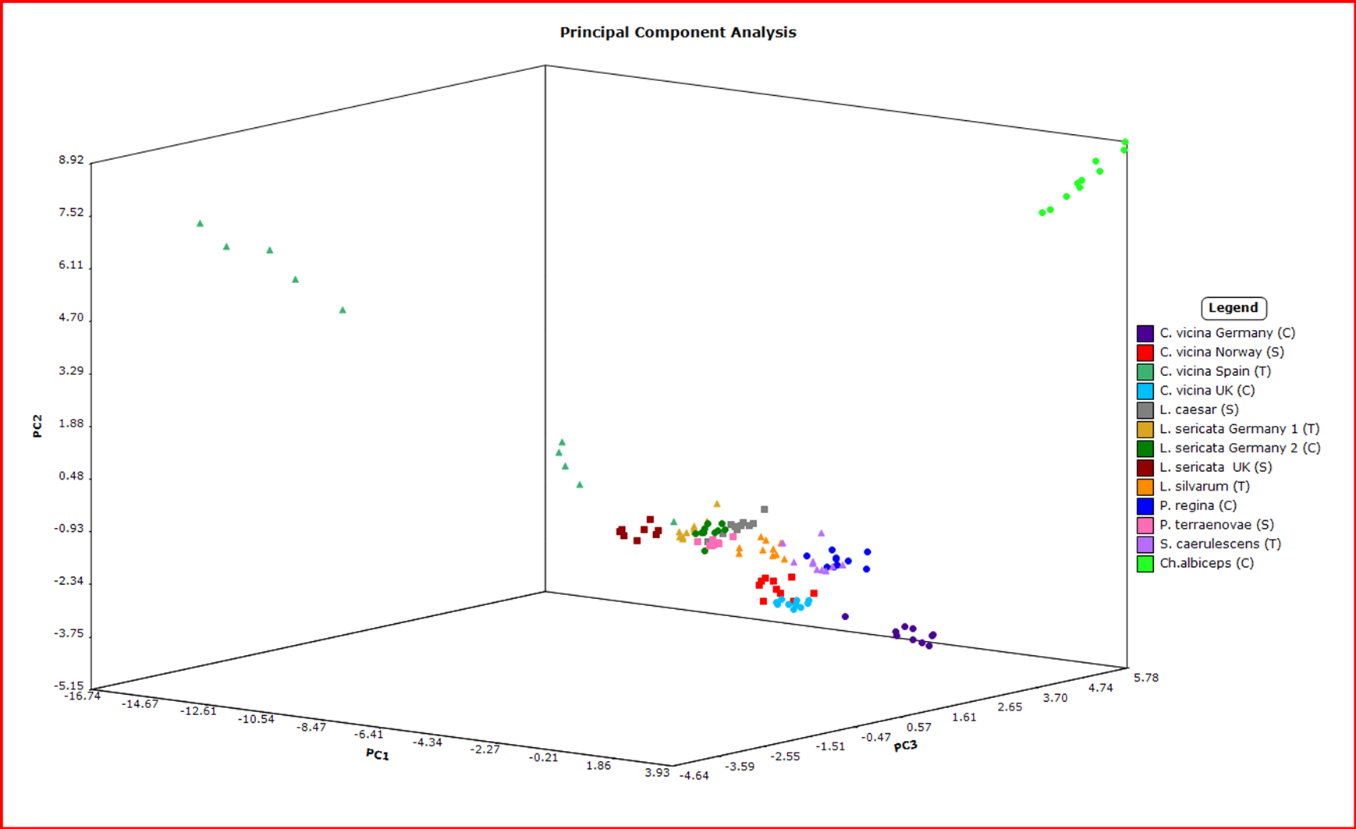
*

Figure S6: Principal component analysis for from the 8 species (thirteen datasets). The compounds used for PCA are given in Table 1. The first three principal components cover 28.2%, 19.3% and 12.7% of the variance respectively for a total of 60.2% variance. Factor loadings for the first two principal components are shown in biplot (Figure S7).

Figure S7. Biplot showing PCA factor loadings for all samples.
